# Painful plaques in a woman with recurrent squamous cell carcinoma

**DOI:** 10.1016/j.jdcr.2023.09.034

**Published:** 2023-10-16

**Authors:** Kyle Seigel, David Croitoru, Orli M. Silverberg, Yvette Miller-Monthrope, Stephane Laframboise, Marissa Joseph

**Affiliations:** a Temerty Faculty of Medicine, University of Toronto, Toronto, Ontario, Canada; b Division of Dermatology, Department of Medicine, University of Toronto, Toronto, Ontario, Canada; c Division of Dermatology, Women’s College Hospital, University of Toronto, Toronto, Ontario, Canada; d Division of Pathology, St. Michael’s Hospital, University of Toronto, Toronto, Ontario, Canada; e Division of Gynecology & Gynecologic Oncology, Princess Margaret Hospital, University of Toronto, Toronto, Ontario, Canada; f Section of Pediatric Dermatology, Department of Pediatrics, The Hospital for Sick Children, University of Toronto, Toronto, Ontario, Canada

**Keywords:** acute febrile neutrophilic dermatosis, erysipeloid Sweet, localized Sweet, paraneoplastic Sweet, Sweet syndrome

## History

**Figure 1:**
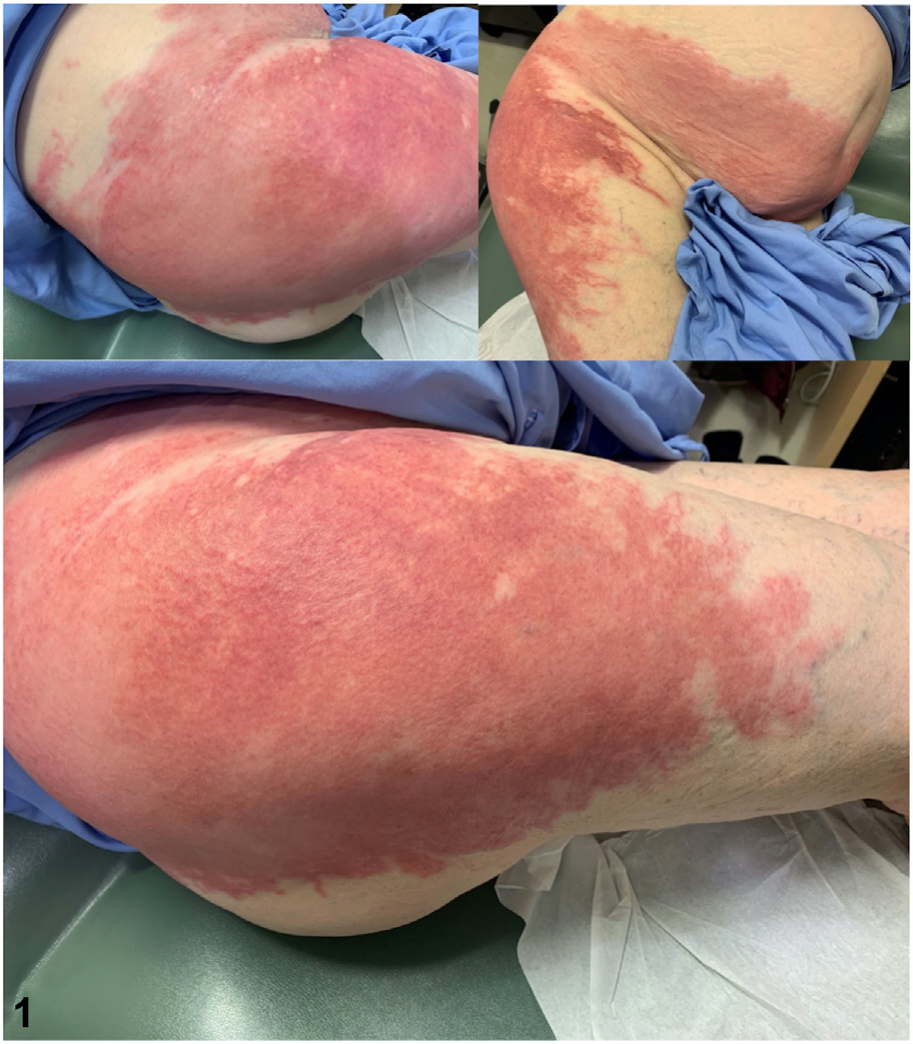

**Figure 2:**
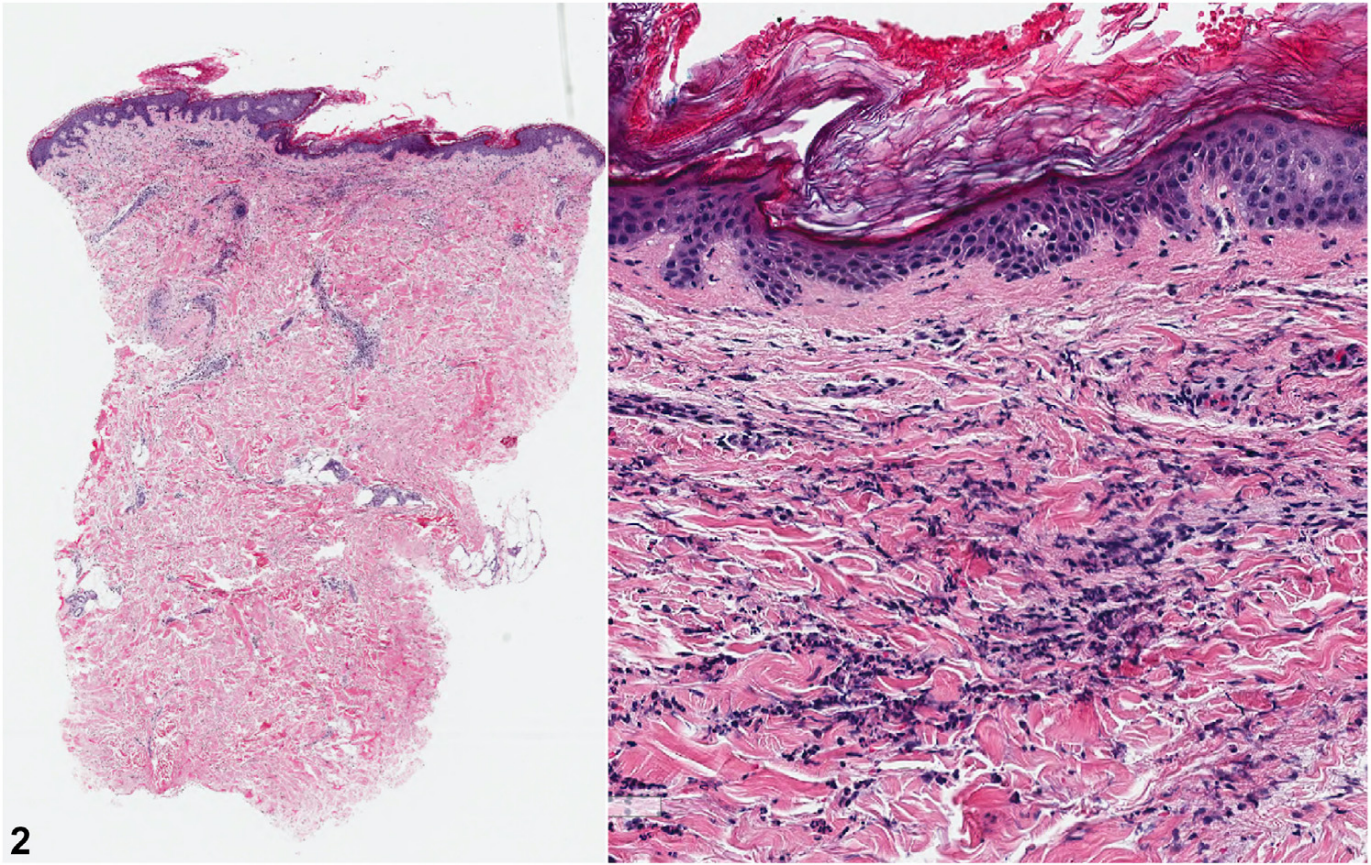

A 79-year-old woman with an elevated body mass index presented with a painful, pruritic, tender plaque on her inferior abdomen, extending to her superior mons pubis and progressing to her right lateral thigh, intermittently recurring with rigors and night sweats for over 6 years (Fig 1). She had a history of vulvar squamous cell carcinoma and vulvar lichen sclerosus since 2013 and was awaiting re-excision for her third squamous cell carcinoma recurrence. None of her medications had been dose-adjusted or introduced within this time frame. Her skin biopsy revealed a dense infiltration of neutrophils with scattered lymphocytes and histiocytes, without evidence of vasculitis or infection (Fig 2). She had an elevated white blood cell count, neutrophil count, and C-reactive protein level, but her serum protein electrophoresis, antinuclear antibody and rheumatoid factor were all within normal limits.

**Question 1: What is the most likely diagnosis?**

A. Pyoderma gangrenosum (PG)
B. Erythema nodosum (EN)
C. Sweet syndrome (SS)
D. Acute inflammatory edema (AIE)
E. Neutrophilic eccrine hidradenitis (NEH)

**Answers:**

A. PG – Incorrect. Although PG is consistent with the histopathology and history of malignancy presented here, PG is more commonly characterized by the presence of nonhealing ulcers, which were not observed in this case.
B. EN – Incorrect. EN typically manifests as tender, erythematous nodules or plaques on the bilateral shins, although it can rarely be observed on the thighs and buttocks, as observed here. Septal panniculitis is typically the predominant feature on EN histopathology.
C. SS – Correct. SS is the most likely diagnosis. This case meets both major diagnostic criteria for SS, including the acute onset of painful erythematous plaques and histologic evidence of a dense neutrophilic infiltrate without vasculitis, as well as all 4 minor criteria, including fever, elevated inflammatory marker levels, appropriate response to systemic treatment and occurrence with an associated disease. More specifically, the widespread and relapsing giant lesions in this patient with an elevated body mass index are most consistent with the giant cellulitis-like variant of SS, as previously described.^1^
D. AIE – Incorrect. AIE is consistent with this case in that it typically involves areas of dependence while sparing those of pressure, with laboratory findings of leukocytosis and neutrophilic infiltrates on histopathology. However, AIE is more commonly bilateral, edematous, and associated with fluid overload.
E. NEH – Incorrect. The association with malignancy is consistent with NEH; however, NEH is more likely to present on the extremities, trunk, or face in patients receiving chemotherapy, and histopathology characteristically reveals neutrophilic infiltration of eccrine glands.

**Question 2: Which of the following is not a characteristic histopathologic feature of this disease?**

A. Papillary dermal edema
B. Dense neutrophilic infiltrate in the upper and mid dermis
C. Leukocytoclasis,
D. Vasculitis,
E. Endothelial swelling

**Answers:**

A. Papillary dermal edema – Incorrect. Massive superficial dermal edema is common in SS.
B. Dense neutrophilic infiltrate in the upper and mid-dermis – Incorrect. This is a characteristic histopathologic feature of SS.
C. Leukocytoclasis – Incorrect. Interstitial leukocytoclastic nuclear debris is typically observed.
D. Vasculitis – Correct. The absence of true vasculitic changes is characteristic of SS.^2^
E. Endothelial swelling – Incorrect. This is a characteristic histopathologic feature of SS.

**Question 3: Which of the following is the most appropriate first-line therapy for this patient?**

A. Oral corticosteroids
B. Topical corticosteroids
C. Biologics
D. Oral retinoids
E. Indomethacin

**Answers:**

A. Oral corticosteroids – Correct. Systemic glucocorticoids are recommended as first-line therapy for adults with extensive cutaneous involvement and systemic symptoms. Dapsone, colchicine, and potassium iodide have also been successfully used as first-line therapies in patients where avoidance of systemic steroids is indicated due to comorbidities or other factors.
B. Topical corticosteroids – Incorrect. There is evidence supporting high-potency topical or intralesional corticosteroids alone in patients with limited SS lesions (less than 5% body surface area) in the absence of systemic symptoms.^3^ However, in patients with more widespread lesions and systemic features, local therapy is favored as an adjunct to systemic steroids.
C. Biologics – Incorrect. Although emerging reports have supported the efficacy of tumor necrosis factor inhibitors, anti-CD20 antibodies, interleukin 12/23 inhibitors, interleukin 1 inhibitors, interleukin 6 inhibitors, and JAK inhibitors in refractory cases of SS,^4^ they would not be recommended as first-line therapies.
D. Oral retinoids – Incorrect. Although oral retinoids have been used successfully in patients with SS recalcitrant to systemic steroids,^5^ they would not be recommended as first-line therapies.
E. Indomethacin – Incorrect. High-dose indomethacin showed benefit in an uncontrolled study of 18 patients with SS^5^ but is not currently recommended as first-line therapy.

## Conflicts of interest

None disclosed.
